# Dr. Jesse S. Myer

**Published:** 1913-12

**Authors:** 


Dr. Jesse S. Myer, University of Missouri, 1893, died at his home in St. Louis, October 30, aged 40. He also held a degree from the Marion Simms College of Medicine, 1896, and had studied at the Charité Hospital of Berlin. Death was due to leucocythæmia. He was a member of the Council of the American Gastro-enterological Association, editor of its Proceedings, and had, on behalf of that organization, published the life and letters of Wm. Beaumont, whose pioneer investigation on the physiology of the stomach in a case of traumatic gastrostomy were, a few years ago, presented to the Association.

